# Affective temperaments, anxiety, and depression as predictors of perioperative pain and analgesic requirements

**DOI:** 10.1192/j.eurpsy.2025.850

**Published:** 2025-08-26

**Authors:** N. Mate-Horvath, N. Acs, X. Gonda

**Affiliations:** 1 Department of Anesthesia and Perioperative Care; 2 Department of Obstetrics and Gynaecology; 3 Department of Psychiatry and Psychotherapy; 4 NAP3.0 Neuropsychopharmacology Research Group, Semmelweis University, Budapest, Hungary

## Abstract

**Introduction:**

Psychological factors, including personality and temperament show an association with various somatic conditions and may also influence perioperative analgesic requirements. Understanding the role of psychological factors could help personalise pain management, improving well-being of patients and reducing postoperative complications. Affective temperaments (AT) have previously been associated with course and characteristics of several somatic conditions, but have not been studied in this context.

**Objectives:**

We aim to investigate the association between ATs and postoperative analgesic needs in a prospective study.

**Methods:**

In our ongoing study we plan to enroll 350 women awaiting non-obsetrical, non-oncological gynecological surgery. Type of surgery and anesthesia are standardized. Psychological evaluation is carried out at three timepoints: 5 days preoperatively (ATs, anxiety, depression, pain catastrophizing)? 24 hours postop (amount and quality of pain experienced, anxiety, depression, current pain catastrophizing); 7 days postop (same as before, plus length of stay and quality of life). Tools include TEMPS-A (ATs), STAI-S and T (anxiety), PHQ9 (depression), PCS (pain catastrophizing) and BPI (pain experience and QoL). Intraoperative vital parameters and opioid use are also observed, as well as the use of postoperative analgesic medications. Statistical associations between pain and psychological factors are analysed using MANOVA, regression, correlation and path analysis.

**Results:**

Currently we are at 50% of our data collection (N=175). At this stage of our research we already have significant preliminary results. Apart from psychological factors, age has a negative predictive effect on pain experienced on day 1 (p<0.001, stβ=-0.327, R2=0.14) and also predicts the effectiveness of painkillers on day 1 (p=0.006, stβ=0.036). When controlled for age ATs also predict pain experienced on day 1: anxious scores predict amount of pain experienced (p=0.049, stβ=0.283), whereas higher dysthymic scores predict smaller amount of pain experienced (p=0.006, stβ=-0.382). Preoperative anxiety and pain expectations do not seem to predict pain, but pain expectations predict how pain effects overall mood (postop day 1 p=0.002, stβ=0.227 and day 7 p=0.035, stβ=0.226). Postop day 7 pain is less influenced by the investigated factors, but state anxiety on postop day 1 predicts postop pain on day 7 (p=0.028, stβ=0.251).

**Conclusions:**

Our study may help in clinical practice to identify patients who are likely to experience more postoperative pain. While age is a significant negative predictor of early and late postoperative pain, when controlling for age, ATs seem to be sensitive predictors of early postoperative pain experience, whereas other investigated factors have a less direct effect on pain experience. Further data analysis with more complex models is needed after data collection is finished.

**Disclosure of Interest:**

None Declared

